# Nanoemulsions With Essential Oils of *Piper* spp. and Their Antifungal and Cytotoxic Potential

**DOI:** 10.1002/cbdv.71614

**Published:** 2026-08-26

**Authors:** Midiã Rodrigues de Oliveira, Roosalyn Santos da Silva, Brendo Pablo Ribeiro da Silva, Marcelo Sousa, Henrique Alves da Silva, Lizandro Manzato, Gemilson Soares Pontes, Antonia Queiroz Lima de Souza, Rita de Cássia Saraiva Nunomura

**Affiliations:** ^1^ Federal University of Amazonas Manaus Brazil; ^2^ Amazonas State University Manaus Brazil; ^3^ National Institute of Amazonian Research Manaus Brazil

**Keywords:** cancer, nanotechnology, pathogenic microorganisms, piperaceae, volatile compounds

## Abstract

Nanoemulsions containing essential oils from *Piper* species remain underexplored for addressing significant public health issues, including cell proliferative disorders and pathogenic microorganisms, highlighting the need to expand research in this field. This study aimed to develop and characterize nanoformulations containing essential oils from *Piper* spp., and to evaluate their safety and efficacy in cell lines, as well as their antimicrobial potential against pathogenic microorganisms. To prepare the nanoemulsions, the essential oils were mixed with Tween 80 in varying ratios (1:1, 1:1.5, 1:2, and 1:2.5). The stability of the nanoformulations was monitored through visual inspection and measurements of particle size, zeta potential, and pH, along with characterization by transmission electron microscopy. Cytotoxic and antimicrobial activities were assessed using colorimetric assays with breast cancer cells (MDA‐MB‐231) and Vero cells (CCL81), as well as pathogenic bacteria and fungi such as *Aspergillus* spp., *Candida* spp., and *Penicillium* sp. The *Piper aduncum* nanoemulsion was the most promising sample, eliminating essential oil toxicity to healthy cells (IC50 >100 µg/mL) and exhibiting the strongest antifungal activity, with MIC values of 125 µg/mL against *Penicillium adametzii* and 250 µg/mL against *Aspergillus flavus*, compared to 1000 µg/mL for the free essential oil. These nanoemulsions remained stable for up to 360 days and had particle sizes ranging from 26.4 ± 0.42 to 265 ± 0.00 nm with zeta potentials between −24.95 ± 0.92 mV and −55.65 ± 0.21 mV. This study demonstrates that nanoemulsions derived from *Piper* spp. are promising candidates against pathogenic microorganisms, with potentials applications in health and food preservation.

## Graphical abstract

The study develops nanoemulsions of *Piper* spp. essential oils, with particle sizes of 26.4–265 nm, zeta potential of −55.65 to −24.95 mV, and stability up to 360 days. The formulations demonstrate cellular safety and antimicrobial efficacy against *Candida* spp., *Penicillium adametzii*, *Aspergilus niger*, and *Aspergilus flavus* under experimental conditions, with potential application in public health and food safety.

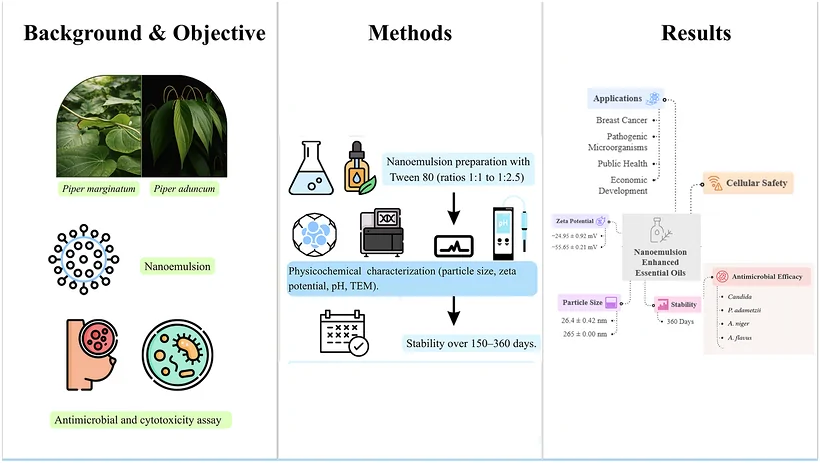

## 1 Introduction

Global public health challenges have led to a significant increase in demand for natural and effective alternatives for disease treatment. In this context, studies have shown that various types of cancer are susceptible to bioactive compounds derived from plants [1]. Similarly, the rise in *Candida* infections resistant to antifungal agents, particularly among cancer patients, underscores the urgent need for novel antifungal therapies [2, 3]. In addition, pathogenic bacteria associated with hospital‐acquired infections and food contamination pose ongoing challenges to global food safety and public health [4]. Likewise, filamentous fungi contribute to food spoilage and cause substantial economic losses [5, 6].

Essential oils are complex mixtures of volatile substances with a wide range of biological activities. However, their hydrophobic nature and volatility often hinder their biotechnological applications. A promising approach to overcome these limitations involves dispersing essential oils in the form of nanoemulsions, a technique that improves the stability and bioavailability of these compounds [7]. Nanoemulsification, which consists of mixing immiscible phases under controlled conditions, has been widely used to incorporate essential oils into food and pharmaceutical products, thereby improving their compatibility with aqueous systems [8].

Rigorous evaluation of the biological and toxicological properties of nanoemulsions is essential to ensure their safety and efficacy in practical applications. Recent studies have demonstrated that nanoemulsification not only enhances the biological activity of essential oils but also reduces their toxicity, making them safer for human and environmental use [9, 10]. In this regard, essential oils from *Piper* species, such as *Piper aduncum*, *Piper hispidum*, and *Piper marginatum*, stand out primarily due to their antimicrobial and cytotoxic effects [11, 12, 13]. Although scientific evidence remains limited, the application of nanoemulsions containing *Piper* spp. oils in health and agriculture is promising, particularly for controlling pathogens and harmful organisms [14, 15].

Considering this scenario, the present study aimed to develop and characterize nanoformulations containing essential oils from *P. aduncum*, *P. hispidum*, and *P. marginatum*, with a comprehensive assessment of their antimicrobial efficacy and cytotoxic profile. This innovative approach seeks not only to address the inherent physicochemical limitations of essential oils but also to explore their therapeutic potential in a safe and efficient manner, offering sustainable alternatives to global health challenges.

## 2 Materials and Methods

### 2.1 Preparation and Characterization of Nanoemulsions

The essential oils were obtained by hydrodistillation of *Piper* species collected in the Manaus‐AM region and characterized by GC‐MS, as reported by Oliveira et al. [16]. Nanoemulsions were prepared according to a methodology adapted from Sugumar et al. [17]. For this purpose, 1% essential oil and varying concentrations of polysorbate 80 (Sigma‐Aldrich, São Paulo, Brazil) were mixed with 10 mL of ultrapure water. The components were homogenized at room temperature at 12,000 rpm for 10 min using an ULTRA‐TURRAX disperser (IKA).

#### 2.1.1 Nanoparticle Stability and pH Determination

Particle size and zeta potential of the nanoemulsions were evaluated by dynamic light scattering (DLS) using a HORIBA SZ‐100 instrument. The samples were diluted in ultrapure water (1:100) and analyzed on days 0, 150, and 360.

The pH of the nanoemulsions was measured using a OneSense pH2500 pH meter. The instrument was initially calibrated with acidic (pH 4.00 ± 0.02) and neutral (pH 7.00 ± 0.02) buffer solutions until 98% measurement accuracy was achieved. Measurements were performed in triplicate.

#### 2.1.2 Transmission Electron Microscopy (TEM)

Nanoemulsion imaging was performed using a JEOL JEM‐2100 transmission electron microscope equipped with a LaB_6_ electron gun, operating at an acceleration voltage of 200 kV and a resolution of 0.25 nm. A drop of each sample was placed onto Formvar/carbon‐coated 200‐mesh grids, and excess liquid was removed with filter paper after 30 s, followed by the addition of a drop of 2% phosphotungstic acid. After an additional 30 s, the samples were dried to form a continuous film and then visualized by TEM.

### 2.2 Antimicrobial Activity

#### 2.2.1 Microorganisms

Bacterial strains used in this study were obtained from the FIOCRUZ‐AM pathogenic microorganism collection: *Staphylococcus aureus* (CBAM 0321), *Escherichia coli* (CBAM 0140), *Pseudomonas aeruginosa* (CBAM 0065), *Enterococcus faecalis* (CBAM 0670), and *Bacillus cereus* (CBAM 0357). Yeasts and filamentous fungi were obtained from the Laboratory of Bioassays and Amazon Microorganisms (LaBMicrA): *Candida glabrata* (LaBMicrA 942), *Candida parapsilosis* (963), *Candida tropicalis* (INCQS 40281), *Penicillium adametzii* (180), *Aspergillus niger* (560), and *Aspergilus flavus* (896).

#### 2.2.2 Determination of Antimicrobial Activity

Cell‐based assays used tetracycline and ampicillin suspensions as positive controls for *E. coli* and *P. aeruginosa*, and for *S. aureus*, *E. faecalis*, and *B. cereus*. Ketoconazole (1000 µg/mL) was used as a positive control for *C. tropicalis*, *C. glabrata*, and *C. parapsilosis*, while polysorbate 80 (2%) served as the negative control. Nanoemulsions (100 µL) were subjected to serial dilutions to obtain concentrations of 5, 2.5, 1.25, 0.62, 0.312, 0.156, and 0.078 mg/mL. Assays were incubated at 36 ± 1°C for 24 h (bacteria) or 48 h (yeasts) and revealed using 2,3,5‐triphenyltetrazolium chloride (TTC).

Assays with filamentous fungi were performed in 12‐well plates containing Sabouraud agar, using samples concentrations of 5, 2.5, 1.25, and 0.62 mg/mL. Ketoconazole was used as the positive control and Tween 80 as the negative control. Mycelial discs of each fungus were inoculated into the wells and incubated for 3–5 days at 30 ± 2°C under photoperiod conditions.

### 2.3 Cytotoxic Activity

Cell proliferation was evaluated using the methylthiazolyldiphenyl‐tetrazolium (MTT) assay, according to the protocol described in Amrati et al. [18]. Briefly, the breast cancer cell line MDA‐MB‐231 (CVCL_0062) and noncancerous Vero CCL81 cells (CVCL_0059) were seeded in 96‐well plates (1 × 10^5^ cells/well) and treated with sample concentrations ranging from 3 to 100 µg/mL. Plates were incubated for 24, 48, and 72 h at 37°C in a humidified atmosphere containing 5% CO_2_. Relative cell viability was calculated using the following equation:

$$\begin{array}{ccc} \% & & \mathrm{Viability}\\ & = & \frac{\mathrm{optical}\;\mathrm{absorbance}\;A570\;\mathrm{of}\;\mathrm{treated}\;\mathrm{sample}}{\mathrm{optical}\;\mathrm{absorbance}\;A570\;\mathrm{of}\;\mathrm{negative}\;\mathrm{control}}\;\times \;100 \end{array}$$

The 50% inhibitory concentration (*IC*_50_) of the samples was calculated using nonlinear regression analysis. Data were analyzed using GraphPad Prism software (v. 8.0). Mann–Whitney or ANOVA tests were employed to assess the relative cytotoxicity of the tested substances.

## 3 Results and Discussion

### 3.1 Development and Characterization of Nanoemulsions

According to Oliveira et al. [16], *P. aduncum* essential oil is rich in dillapiole (80.26 %). The authors also reported the presence of *β*‐selinene (17.77%), (*E*)‐*iso*‐*γ*‐bisabolene (17.71%) e *γ*‐terpinene (12.75%) in *P. hispidum*; and (*E*)‐*iso*‐*γ*‐bisabolene (26.85%) in *P. marginatum* essential oils. Other studies on *Piper* spp. essential oils have demonstrated biological activities such as insecticidal and acaricidal effects, as well as notable antimicrobial potential against pathogenic microorganisms [12, 13, 15, 19], with these effects being closely related to the chemical composition of these natural products.

Macroscopic analyses revealed that the most stable nanoemulsions were those formulated with essential oils from *P. aduncum* (PAN) and *P. marginatum* (PMN) at a 1:2 oil‐to‐surfactant ratio, which exhibited a translucent appearance. In contrast, nanoemulsions prepared with *P. hispidum* essential oil (PHN) changed from translucent to colorless after 15 days of observation, indicating a potential loss of stability. According Shukla and Singh [20], this issue may be addressed by adjusting the surfactant concentration or by adding or substituting another surfactant.

#### 3.1.1 Nanoparticle Stability and pH

Analyses of particle size, polydispersity index, and zeta potential of the samples (Table 1) showed variations over time, confirming that only *P. marginatum* and *P. aduncum* nanoemulsions formulated at the 1:2 oil‐to‐surfactant ratio remained stable over extended periods (Table 1), corroborating the macroscopic observations. The initial particle size and polydispersity index observed for PAN are comparable to those reported by Swanthy et al. [14] for *P. nigrum* essential oil nanoemulsions prepared with Tween 80 at the same 1:2 ratio. However, while the *P. nigrum* nanoemulsion degraded after 30 days of storage, PAN and PMN remained stable for more than 150 and 360 days, respectively. This enhanced stability may be attributed to the higher zeta potential values obtained for PAN and PMN compared with those reported for *P. nigrum* nanoemulsions developed by Swanthy et al. [14].

**TABLE 1:** Physicochemical parameters of *Piper* spp. nanoemulsions at a 1:2 oil‐to‐surfactant ratio.

Sample	pH	Day	Size (nm)	Zeta (mV)	PDI
PAN	3.98	1	26.4 ± 0.42	−34.80 ± 1.84	0.14 ± 0.5
		150	86.75 ± 0.07	−29.5 ± 3.29	0.24 ± 0.01
		360	89.25 ± 1.20	−28.50 ± 1.27	0.10 ± 0.02
PMN	3.92	1	238 ± 0.00	−55.65 ± 0.21	0.33 ± 0.05
		150	265 ± 0.00	−41.30 ± 042	0.40 ± 0.05
PHN	3.76	1	124.4 ± 0.14	−24.95 ± 0.92	0.36 ± 0.04

Abbreviations: PDI: Polydispersity Index. PAN: *P. aduncum* nanoemulsion. PMN: *P. marginatum* nanoemulsion. PHN: *P. hispidum* nanoemulsion.

#### 3.1.2 Transmission Electron Microscopy (TEM)

TEM analyses were performed only for PAN and PMN, as these nanoemulsions remained stable for the longest period (Table 1). The electron micrographs revealed the morphology, size, and distribution of essential oil droplets within the nanoformulations.

**FIGURE 1:**
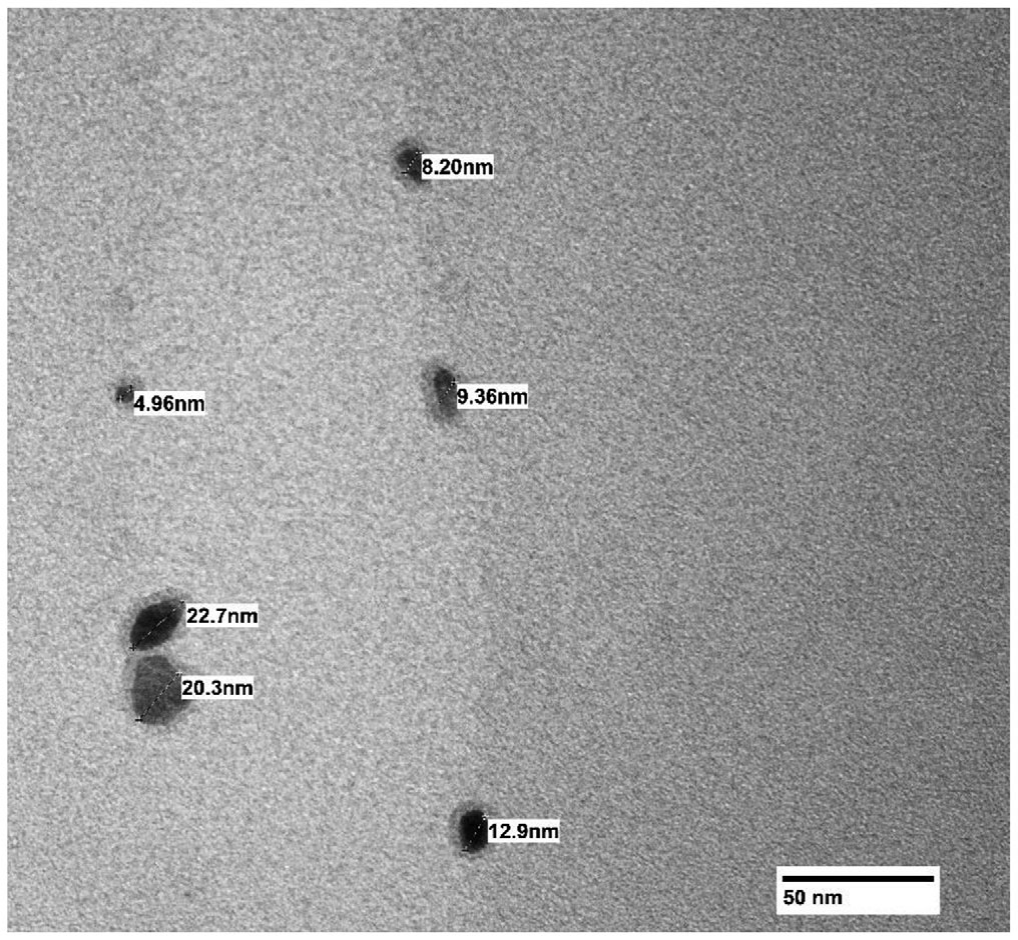
Transmission electron micrograph of *P. aduncum* nanoparticles (PAN).

Figure 1 illustrates the uniform distribution and ellipsoidal shape of essential oil droplets dispersed in the *P. aduncum* nanoemulsion, with sizes ranging from 4.96 to 22.7 nm. These findings corroborate the DLS measurements obtained for the freshly prepared sample, as presented in Table 1. Moreover, the homogeneous particle distribution reflects good surfactant compatibility with the formulation, as supported by the zeta potential values. Similar results were reported by Vinh et al. [21] for a *P. nigrum* essential oil nanoemulsion prepared with Tween 80 using a more costly technique.

**FIGURE 2:**
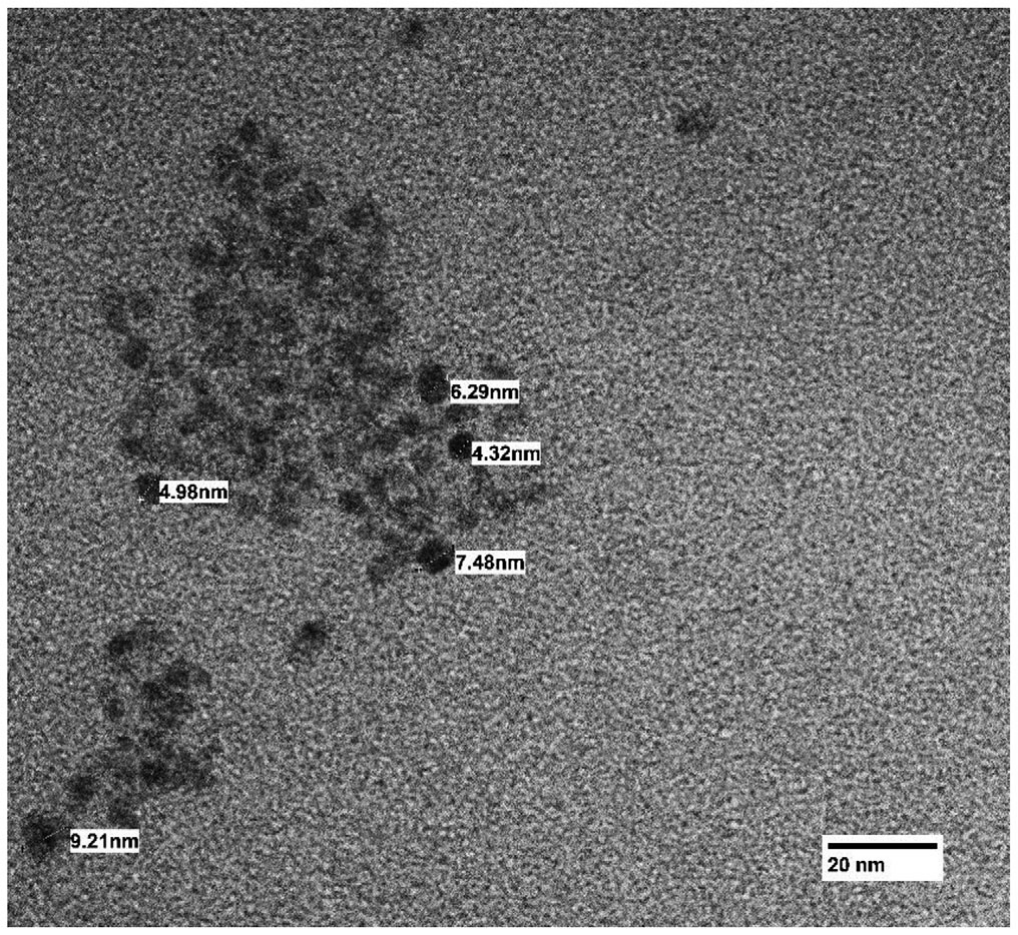
Transmission electron micrograph of *P. marginatum* nanoparticles (PMN).

Figure 2 shows the morphology of lipid nanoparticles in the *P. marginatum* nanoemulsion, revealing predominantly spherical particles. Some regions of the sample exhibited particle aggregates, with sizes ranging from 4.98 to 9.21 nm. However, visual inspection did not indicate flocculation or increased creaminess in this formulation. According to Shukla and Singh [20] and Vinh et al. [21], the low occurrence of aggregates, allied with the absence of physicochemical changes, does not compromise the stability or biological efficacy of the nanoemulsions.

### 3.2 Antimicrobial Activity

The essential oils of *Piper* spp. previously evaluated by Oliveira et al. [16] did not exhibit activity against the bacteria investigated, nor did the nanoemulsions. However, some of these samples were active against fungi and yeasts, with distinct potencies depending on the formulation and the target species (Table 2).

**TABLE 2:** *Piper* spp. essential oils and nanoemulsions with antimicrobial activity.

Microorganisms	PAEO	PAN	PHEO	PMN	C+
*C. glabrata*	++250 µg/mL			+500 µg/mL	++15.62 µg/mL
*C. tropicalis*				+500 µg/mL	++31.25 µg/mL
*C. parapsilosis*	++125 µg/mL				++7.81 µg/mL
*P. adametzii*	+1000 µg/mL	+125 µg/mL			++125 µg/mL
*A. niger*	+1000 µg/mL	+1000 µg/mL		+250 µg/mL	+500 µg/mL
*A. flavus*		+250 µg/mL	+1000 µg/mL		+500 µg/mL

Fungistatic (+); fungicidal (++).

Abbreviations: PAEO: *P. aduncum* essential oil. PAN: *P. aduncum* nanoemulsion. PHEO: *P. hispidum* essential oil. PMN: *P. marginatum* nanoemulsion. C+: positive control. Note: essential oil data were retrieved from Oliveira et al. [16].

The *P. aduncum* essential oil (PAEO) showed fungicidal activity against *C. glabrata* (MIC 250 µg/mL) and *C. parapsilosis* (MIC 125 µg/mL), as well as fungistatic activity against *P. adametzii* and *A. niger* (MIC 1000 µg/mL). While the corresponding nanoemulsion (PAN) demonstrated enhanced fungistatic effects against *P. adametzii* (MIC 125 µg/mL), *A. flavus* (MIC 250 µg/mL), and *A. niger* (MIC 1000 µg/mL).

The nanoemulsion disperses the essential oil into nanometric droplets, which can result in increased bioavailability of the antimicrobial compound [22]. This may explain the absence of antimicrobial activity for the essential oil of *P. marginatum* (PMEO) while its derived nanoemulsion (PMN) exhibited fungistatic activity against *C. tropicalis* and *C. glabrata*, with an MIC of 500 µg/mL for both yeasts. The nanoformulation also showed fungistatic activity against *A. niger* (MIC 250 µg/mL), indicating a broad antifungal spectrum. In contrast, the *P. hispidum* essential oil (PHEO) exhibited activity exclusively against *A. flavus* (MIC 1000 µg/mL), while its corresponding nanoemulsion showed no antimicrobial activity. The interaction between the *P. hispidum* essential oil and the nanoemulsion surfactant may have compromised the availability of the active ingredient, resulting in its release at concentrations below the levels required for microbial inhibition [23].

These results corroborate previous studies reporting the antifungal activity of *Piper* spp. essential oils and nanoemulsions [14, 24]. The observed antifungal efficacy of certain samples may be associated with increased solubility and bioavailability of active constituents promoted by nanoemulsification [20, 25, 26]. These results reinforce the potential of *Piper* essential oil nanoemulsions in controlling mycotoxin‐producing fungal strains and pathogenic yeasts.

### 3.3 Cytotoxic Activity

Only samples that reduced cell viability below 80% at 72 h were considered cytotoxic. The cytotoxicity of *Piper* spp. essential oils in Vero CCL‐81 cells was previously described in Oliveira et al. [16]. Compared to these findings, nanoemulsification enhanced the cellular safety of these oils in the Vero lineage without compromising their antiproliferative activity against MDA‐MB‐231 under the experimental conditions tested.

**FIGURE 3:**
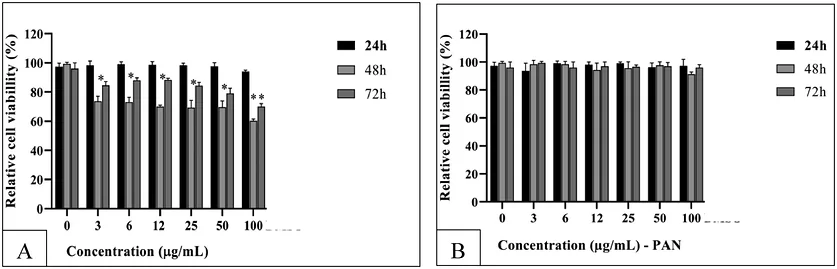
Cytotoxicity assay results against CCL81 noncancerous cells for: (A) *P. aduncum* essential oil (PAEO) and (B) *P. aduncum* nanoemulsion (PAN).

**FIGURE 4:**
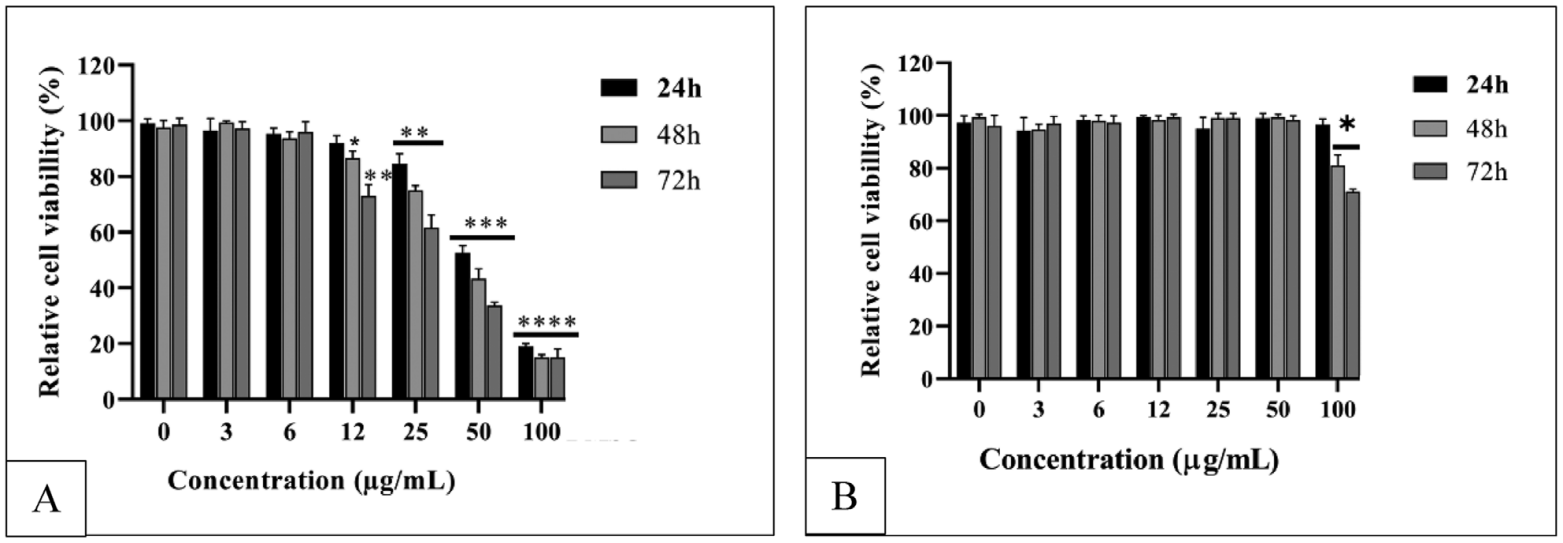
Cytotoxicity assay against CCL81 noncancerous cells for: (A) *P. hispidum* essential oil (PHEO) and (B) *P. hispidum* nanoemulsion (PHN).

**FIGURE 5:**
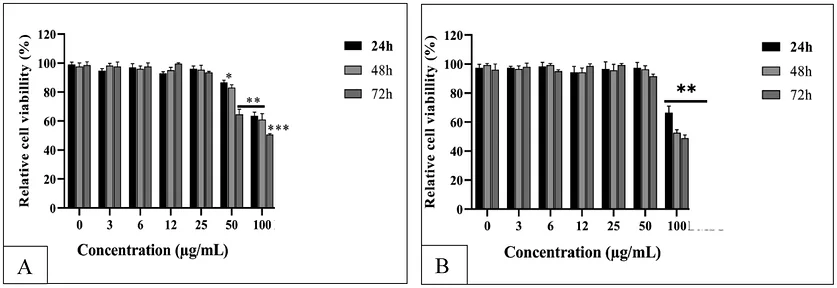
Cytotoxicity assay results against CCL81 noncancerous cells for: (A) *P. marginatum* essential oil (PMEO) and (B) *P. marginatum* nanoemulsion (PMN).

The best result was obtained for the *P. aduncum* nanoemulsion, which eliminated the toxicity of the essential oil on healthy Vero cells at all concentrations tested (Figure 3). A similar outcome was observed for the *P. hispidum* nanoemulsion, with reduced cytotoxicity (IC_50_> 100 µg/mL) compared to the free essential oil (*IC*_50_ = 23.84 µg/mL) after 72 h of treatment (Figure 4). For *P. marginatum*, the increase in safe concentration on Vero cells was from 25  (essential oil) to 50 µg/mL (nanoemulsion) at 72 h of exposure (Figure 5). The antiproliferative potential of the nanoemulsions against MDA‐MB‐231 did not surpass that of the essential oils, except for the *P. aduncum* nanoemulsion, which reduced cell viability from 6 µg/mL onward after 72 h.

The nanoemulsification of *P. aduncum*, *P. marginatum*, and *P. hispidum* essential oils selectively attenuated toxicity toward healthy Vero cells, increasing safe concentrations by at least twofold under the tested conditions. This enhanced safety profile supports nanoemulsification as a viable strategy for the development of natural product–based pharmaceutical technologies [27]. Moreover, recent studies have reported that essential oil nanoemulsions can enhance cellular penetration, improve the oxidative stability of compounds, and promote sustained release of active constituents, which may explain the greater efficacy observed for the nanoemulsion compared with the crude essential oil in CCL81 cells [28].

## 4 Conclusions

Nanoemulsification of the essential oils of *Piper* spp. reduces cytotoxicity on healthy cells and amplifies the antifungal effect of these samples of *C. tropicalis, C. glabrata, P. adametzii, A. niger*, and *A. flavus*. These nanoemulsions demonstrated excellent physicochemical properties and remained stable over extended periods. Overall, this study demonstrates that nanoemulsions derived from *Piper* spp. are promising candidates for combating pathogenic microorganisms, contributing to advancements in health and food‐related applications.

## Author Contributions

**Midiã Rodrigues de Oliveira, Gemilson Soares Pontes, Antonia Queiroz Lima de Souza, Rita de Cássia Saraiva Nunomura**: conceptualization—ideas, formulation or evolution of overarching research goals and aims. **Midiã Rodrigues de Oliveira, Brendo Pablo Ribeiro da Silva, Gemilson Soares Pontes**: formal analysis**—**application of statistical, mathematical, computational, or other formal techniques to analyze or synthesize study data. **Midiã Rodrigues de Oliveira, Roosalyn Santos da Silva, Brendo Pablo Ribeiro da Silva, Marcelo Sousa, Henrique Alves da Silva, Lizandro Manzato**: investigation—conducting a research and investigation process, specifically performing the experiments, or data/evidence collection. **Marcelo Sousa, Lizandro Manzato, Gemilson Soares Pontes, Antonia Queiroz Lima de Souza, Rita de Cássia Saraiva Nunomura**: methodology—development or design of methodology; creation of models. **Antonia Queiroz Lima de Souza, Rita de Cássia Saraiva Nunomura**: project administration**—**management and coordination responsibility for the research activity planning and execution. **Lizandro Manzato, Gemilson Soares Pontes, Antonia Queiroz Lima de Souza, Rita de Cássia Saraiva Nunomura**: resources—provision of study materials, reagents, materials, patients, laboratory samples, animals, instrumentation, computing resources, or other analysis tools. **Henrique Alves da Silva, Gemilson Soares Pontes, Rita de Cássia Saraiva Nunomura**: supervision**—**oversight and leadership responsibility for the research activity planning and execution, including mentorship external to the core team. **Midiã Rodrigues de Oliveira, Marcelo Sousa, Henrique Alves da Silva, Gemilson Soares Pontes, Antonia Queiroz Lima de Souza, Rita de Cássia Saraiva Nunomura**: validation**—**verification, whether as a part of the activity or separate, of the overall replication/reproducibility of results/experiments and other research outputs. **Midiã Rodrigues de Oliveira, Roosalyn Santos da Silva**: visualization—preparation, creation and/or presentation of the published work, specifically visualization/data presentation. **Midiã Rodrigues de Oliveira, Roosalyn Santos da Silva**: writing – original draft—preparation, creation and/or presentation of the published work, specifically writing the initial draft (including substantive translation). **Roosalyn Santos da Silva, Marcelo Sousa, Antonia Queiroz Lima de Souza, Rita de Cássia Saraiva Nunomura**: writing – review and editing—preparation, creation and/or presentation of the published work by those from the original research group, specifically critical review, commentary or revision—including pre‐ or post‐publication stages.

## Funding

Coordination for the Improvement of Higher Education Personnel, Fundação de Amparo à Pesquisa do Estado do Amazonas (Grant Numbers: 013/2022), Financier of Studies and Projects *Organization*, National Council for Scientific and Technological Development.

## Conflicts of Interest

The authors declare no conflicts of interest.

## Data Availability

The data that support the findings of this study are openly available in Scholar one at https://support.silverchair.com/scholarone‐manuscripts/authors/.

## References

[1] S. Safe , “Natural Products as Anticancer Agents and Enhancing Their Efficacy by a Mechanism‐Based Precision Approach,” Exploration of Drug Science 2 (2024): 408–427, 10.37349/eds.2024.00054.

[2] A. Mesini , M. Mikulska , D. R. Giacobbe , et al., “Changing Epidemiology of Candidaemia: Increase in Fluconazole‐Resistant *Candida parapsilosis* ,” Mycoses 63 (2020): 361–368, 10.1111/myc.13050. 31954083

[3] D. Farmakiotis , J. J. Tarrand , and D. P. Kontoyiannis , “Drug‐Resistant *Candida glabrata* Infection in Cancer Patients,” Emerging Infectious Diseases 20 (2014): 1833–1840, 10.3201/eid2011.140685. 25340258 PMC4214312

[4] WHO , World Health Organization. Global Antimicrobial Resistance and Use Surveillance System (GLASS) Report 2021, Geneva, (2021).

[5] A. B. Snyder and R. W. Worobo , “Fungal Spoilage in Food Processing,” Journal of Food Protection 81 (2018): 1035–1040, 10.4315/0362-028X.JFP-18-031. 29768029

[6] A. Rodríguez , M. Rodríguez , M. J. Andrade , and J. J. Córdoba , “Detection of Filamentous Fungi in Foods,” Current Opinion in Food Science 5 (2015): 36–42, 10.1016/j.cofs.2015.07.007.

[7] P. Farshi , M. Tabibiazar , M. Ghorbani , and H. Hamishehkar , “Evaluation of Antioxidant Activity and Cytotoxicity of Cumin Seed Oil Nanoemulsion Stabilized by Sodium Caseinate‐Guar Gum,” Pharmaceutical Sciences 23 (2017): 293–300, 10.15171/PS.2017.43.

[8] J. N. Haro‐González , B. N. Schlienger De Alba , M. Martínez‐Velázquez , G. A. Castillo‐Herrera , and H. Espinosa‐Andrews , “Optimization of Clove Oil Nanoemulsions: Evaluation of Antioxidant, Antimicrobial, and Anticancer Properties,” Colloids and Interfaces 7 (2023): 64, 10.3390/colloids7040064.

[9] S. Maisanaba , S. Pichardo , M. Jordá‐Beneyto , S. Aucejo , A. M. Cameán , and Á. Jos , “Cytotoxicity and Mutagenicity Studies on Migration Extracts From Nanocomposites With Potential Use in Food Packaging,” Food and Chemical Toxicology 66 (2014): 366–372, 10.1016/j.fct.2014.02.011. 24530314

[10] A. P. Worth , “Types of Toxicity and Applications of Toxicity Testing,” in The History of Alternative Test Methods in Toxicology (Academic Press, 2019), 7–10, 10.1016/B978-0-12-813697-3.00002-0.

[11] O. O. Ferreira , J. N. Cruz , Â. A. B. De Moraes , et al., “Essential Oil of the Plants Growing in the Brazilian Amazon: Chemical Composition, Antioxidants, and Biological Applications,” Molecules 27 (2022): 4373, 10.3390/molecules27144373. 35889245 PMC9318482

[12] M. Rodrigues De Oliveira , L. Anjos Da Silva , R. Santos Da Silva , C. C. Branco De Queiroz , and R. Takeara , “Chemical Composition and Biological Activities of Essential Oils of *Piper* Species From the Amazon,” Journal of Essential Oil Research 33 (2021): 536–548, 10.1080/10412905.2021.1942250.

[13] Ê. S. Carvalho , V. F. S. Ayres , M. R. Oliveira , et al., “Anticariogenic Activity of Three Essential Oils From Brazilian Piperaceae,” Pharmaceuticals 15 (2022): 972, 10.3390/ph15080972. 36015120 PMC9416246

[14] J. S. Swanthy , P. Mishra , J. Thomas , et al., “Antimicrobial Potency of High‐Energy Emulsified Black Pepper Oil Nanoemulsion Against Aquaculture Pathogen,” Aquaculture 491 (2018): 210–220.

[15] A. C. De Oliveira , I. S. C. Sá , R. S. Mesquita , et al., “Nanoemulsion Loaded With Volatile Oil From *Piper Alatipetiolatum* as an Alternative Agent in the Control of *Aedes aegypti* ,” Revista Brasileira de Farmacognosia 30 (2020): 667–677, 10.1007/s43450-020-00092-8.

[16] M. R. Oliveira , E. D. Alfaia Neto , and E. E. C. Alves , “Antioxidant and Cytotoxic Properties of *Piper* spp. Volatile Oils That Inhibit Pathogenic Microorganisms,” Flavour and Fragrance Journal 41 (2026): 935–942.

[17] S. Sugumar , S. K. Clarke , M. J. Nirmala , B. K. Tyagi , A. Mukherjee , and N. Chandrasekaran , “Nanoemulsion of *Eucalyptus* Oil and Its Larvicidal Activity Against *Culex quinquefasciatus* ,” Bulletin of Entomological Research 104 (2014): 393–402, 10.1017/S0007485313000710. 24401169

[18] F. E.‐Z. Amrati , M. Chebaibi , R. Galvão De Azevedo , et al., “Phenolic Composition, Wound Healing, Antinociceptive, and Anticancer Effects of *Caralluma europaea* Extracts,” Molecules 28 (2023): 1780, 10.3390/molecules28041780. 36838767 PMC9961855

[19] V. F. S. Ayres , M. R. Oliveira , C. C. B. Queiroz , et al., “Chemical Composition and Acaricidal Activity of the Essential Oils of *Piper marginatum* and *Piper callosum* Collected in the Amazon Region,” Journal of Essential Oil Research 35 (2022): 1–9.

[20] P. Shukla and S. Singh , “A Complete Review on Nanoemulsion,” Indo American Journal of Pharmaceutical Science 9 (2022): 266–272.

[21] T. D. T. Vinh , L. T. M. Hien , and D. T. A. Dao , “Formulation of Black Pepper (Piper Nigrum L.) Essential Oil Nano‐Emulsion via Phase Inversion Temperature Method,” Food Science & Nutrition 8 (2020): 1741–1752, 10.1002/fsn3.1422. 32328240 PMC7174231

[22] R. D. Dos Santos , B. N. Matos , D. O. Freire , et al., “Chemical Characterization and Antimicrobial Activity of Essential Oils and Nanoemulsions of *Eugenia uniflora* and *Psidium guajava* ,” Antibiotics 14 (2025): 93, 10.3390/antibiotics14010093. 39858378 PMC11763297

[23] F. Donsì and G. Ferrari , “Essential Oil Nanoemulsions as Antimicrobial Agents in Food,” Journal of Biotechnology 233 (2016): 106–120, 10.1016/j.jbiotec.2016.07.005. 27416793

[24] L. S. Silva , J. M. Mar , S. G. Azevedo , et al., “Encapsulation of *Piper aduncum* and *Piper hispidinervum* Essential Oils in Gelatin Nanoparticles: A Possible Sustainable Control Tool of *Aedes aegypti*, *Tetranychus urticae* and *Cerataphis lataniae* ,” Journal of the Science of Food and Agriculture 99 (2018): 685–695, 10.1002/jsfa.9233. 29971785

[25] L. Ruiz‐Vásquez , L. Ruiz Mesia , H. D. Caballero Ceferino , et al., “Antifungal and Herbicidal Potential of *Piper* Essential Oils From the Peruvian Amazonia,” Plants 11 (2022): 1793, 10.3390/plants11141793. 35890427 PMC9324010

[26] Nurmansyah , H. Idris , E. Suryani , H. Gustia , and A. I. Ramadhan , “The Effect of Various Essential Oil and Solvent Additives on the Botanical Pesticide of *Piper aduncum* Essential Oil on Formulation Antifungal Activity,” Results in Engineering 16 (2022): 100644, 10.1016/j.rineng.2022.100644.

[27] W. L. O. Leite and A. A. Oliveira Filho , “Avaliação Farmacológica de Produtos Naturais no Combate Ao Câncer,” Revista Interdisciplinar em Saúde 1 (2014): 192–211.

[28] K. Khairan , M. Mar'ah , and I. Rinaldi , “An Overview of Essential Oil‐Based Nanoemulsion and Their Biological Activities Against Some Microbial Pathogenic,” IOP Conference Series: Earth and Environmental Science 1297 (2024): e012083.

